# Supplementary material for: Scavenging vs hunting affects behavioral traits of an opportunistic carnivore
Source: PeerJ. 2022 May 2;10:e13366. doi: 10.7717/peerj.13366 (PMC9070321; doi:10.7717/peerj.13366)
Supplement: Supplemental Information 1 [file peerj-10-13366-s001.doc]

**Supplementary Information**

Study Subject Information

Our study was conducted on eight coyote pairs, four treatment pairs and four control pairs, ranging from 1.5 to 3.5 years old at the start of the study. Pairs were selected based on logistics; we had to exclude pairs already being used on other studies or previously exposed to objects or tests that were like this experiment and included pairs already slated for reproduction in the group that was allowed to reproduce. We split coyotes equally among treatment groups based on age. Because our study took place across a year, two pairs of treatment coyotes and two pairs of control coyotes reared pups (April – June) during this study for colony management purposes.

Most of the captive coyotes are born on-site to captive parents and left with the parental pair until ~10-weeks old. Facility staff then separate the litter from their parents for 5-10 weeks. They may remain with all their siblings or split into two groups, pending colony space and litter size, with approximately three pups per litter housed together in an enclosure during this phase. Coyotes are paired with their selected mate from another litter at 3-4 months old and remain in this pairing through their adult life. This approach mimics coyote behavior in the wild, with coyotes dispersing at a young age and forming monogamous pair bonds (Hennessy, Dubach & Gehrt, 2012). Coyotes in this facility display similar behavioral budgets to wild coyotes (Shivik et al., 2009). All coyotes used in this study were captive-born with minimal human intervention at the facility. Seven coyotes had entirely captive-born parents and grandparents. Five coyotes had two captive-born parents but at least one wild-born grandparent. Three coyotes had only one captive-born parent and one coyote had two wild-born parents. All the coyotes used in this study had been housed with their mate for >1 year at the start of the study.

For colony management purposes and to facilitate other ongoing research at the facility, coyote pairs were moved to new enclosures 4-6 times throughout the study period. At all times, pairs were housed in enclosures where they could observe 1-3 other study pairs. Previous research at this facility has documented social learning in extractive foraging tasks (Young, Touzot & Brummer, 2019). However, due to the low sample size and limited participation in research tasks, we were unable to test for the potential effects of neighbor pairs.

Coyotes at the facility are minimally handled, and our procedures did not increase handling requirements. All coyotes at the facility are fed six days per week, and we did not alter the feeding schedule during this study. Coyotes that failed foraging tasks on the testing days were fed their regular ration later in the day. We documented instances of intra-pair aggression throughout the study. These interactions were rare and did not change in frequency in response to research procedures.

**Literature Cited**

Hennessy CA, Dubach J, Gehrt SD. 2012. Long-term pair bonding and genetic evidence for monogamy among urban coyotes (*Canis latrans*). *Journal of Mammalogy* 93:732–742. DOI: 10.1644/11-mamm-a-184.1.

Shivik JA, Palmer GL, Gese EM, Osthaus B. 2009. Captive coyotes compared to their counterparts in the wild: does environmental enrichment help? *Journal of Applied Animal Welfare Science* 12:223–235. DOI: 10.1080/10888700902955989.

Young JK, Touzot L, Brummer SP. 2019. Persistence and conspecific observations improve problem-solving abilities of coyotes. *Plos One* 14:e0218778. DOI: 10.1371/journal.pone.0218778.


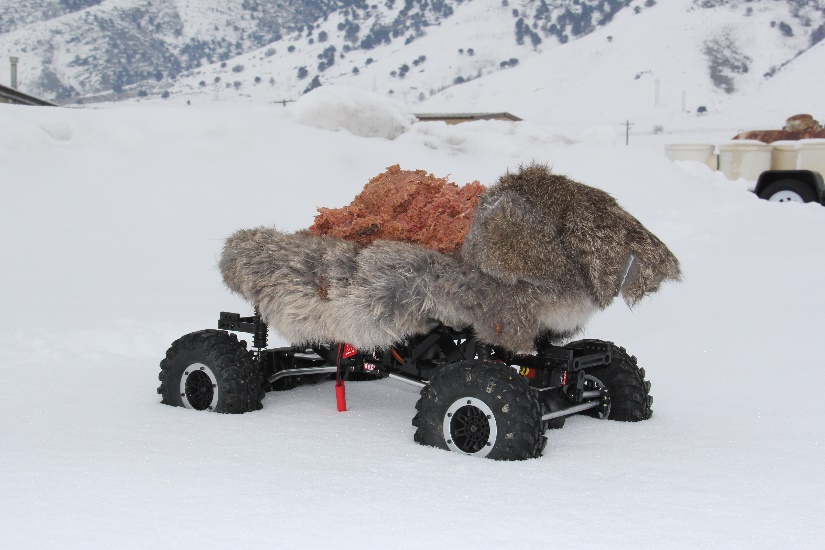


Figure S1. Rabbit-like prey model presented to captive coyotes throughout the study with one portion of food on top. The model was stagnant for control animals but remotely controlled to move for treatment animals to pursue.


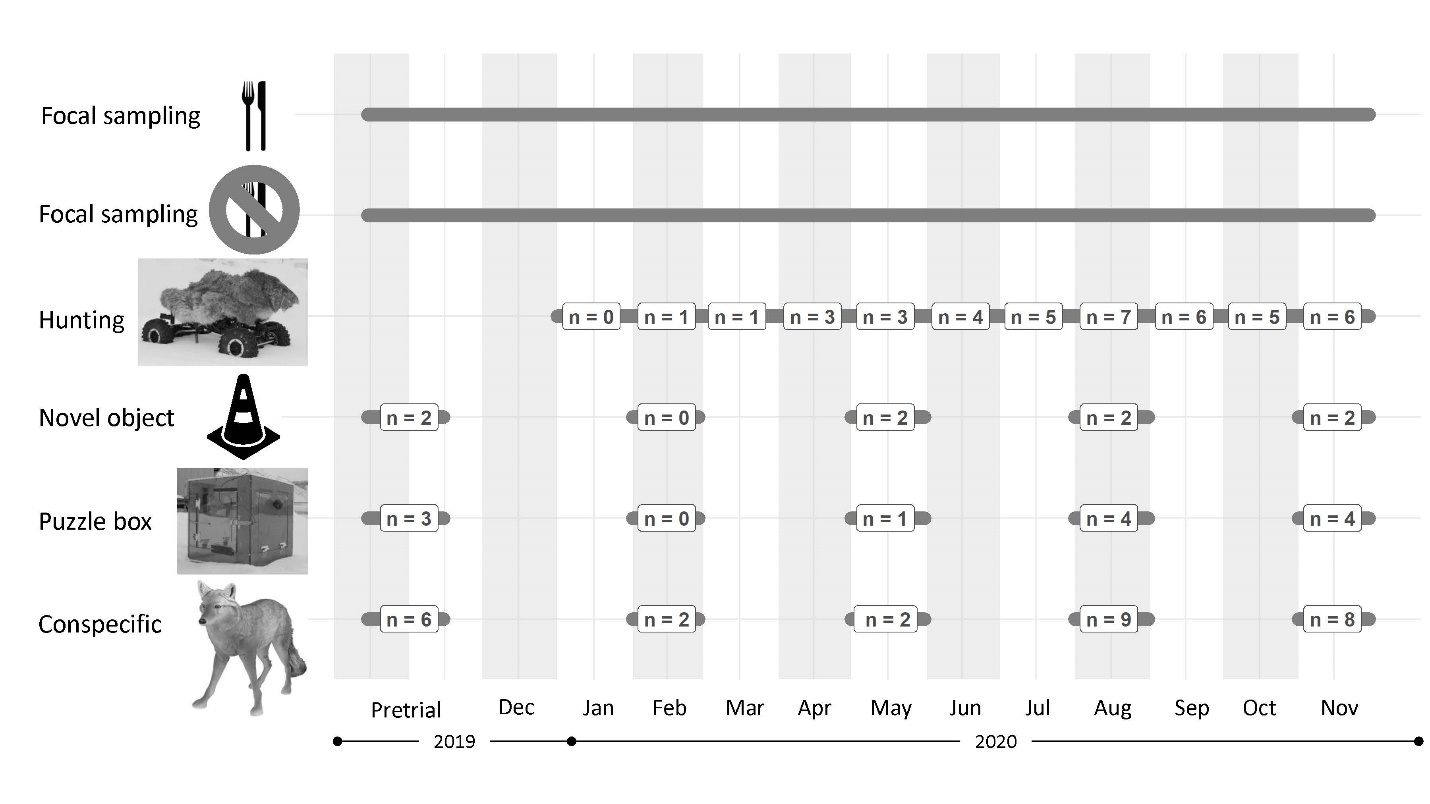


Figure S2. A timeline of research activities indicating when each behavioral test was completed. Focal sampling occurred during feeding and nonfeeding times in all months. The text label indicates the number of coyotes that interacted with the test during each presentation. Hunting the prey model occurred monthly and the label indicates the number of individuals that investigated or interacted with the prey model each month (from n = 16 that were presented with the model). Novel object, puzzle box, and conspecific tests occurred every three months and the label indicates the number of individuals that approached within 1m of the novel object, interacted with the puzzle box, and approached within 5m of the conspecific dummy (from n = 16 individuals).


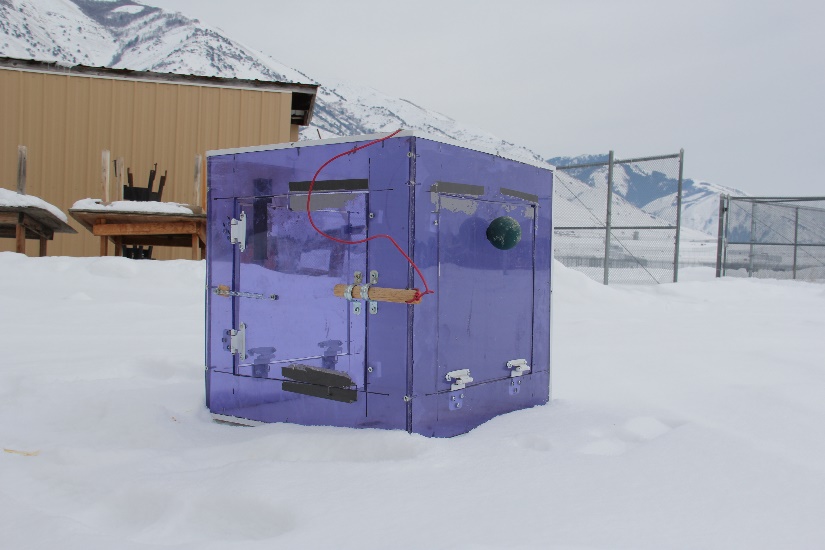


A


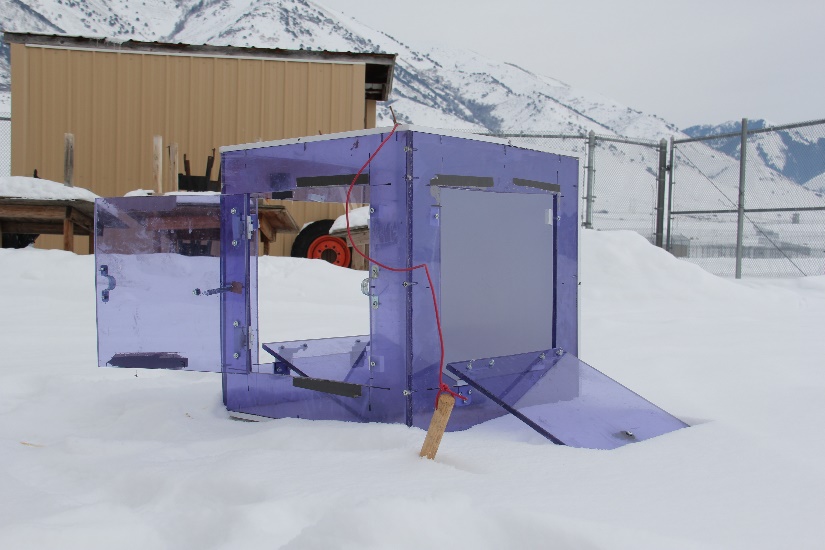


B

Figure S3. A multi-access puzzle box shown with all doors closed (A) and open (B). From left to right, the doors push in, swing open when the wooden peg is removed, and pull out respectively.


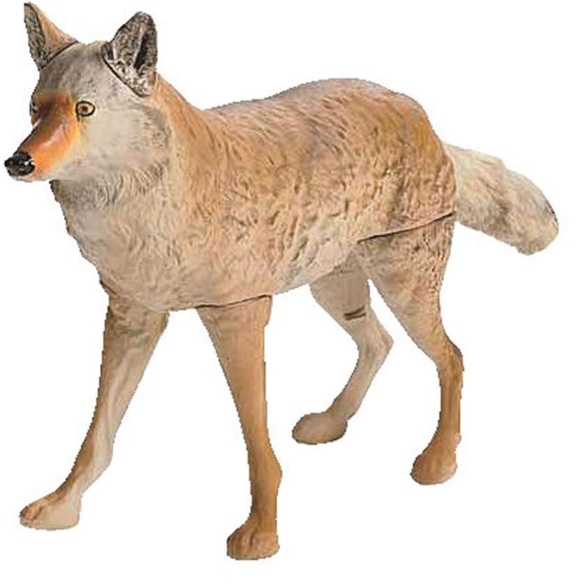


Figure S4. The coyote decoy used for the unknown conspecific behavioral test. Lone Howler Coyote Decoy, Flambeau Outdoors, Middlefield, OH, USA

**Table S1:**

**Definitions and metrics for boldness, innovation, and persistence in our study.**

| Behavioral Trait | Definition | Measure |
| --- | --- | --- |
| Boldness | Individual's response to a potentially risky stimulus | Latency to approach novel object |
| Innovation | Individual's ability to display new behaviors or modify existing behaviors to solve novel problems | Number of puzzle box doors solved |
| Persistence | Amount of time an individual spends interacting with a stimulus | Proportion of time spent interacting with the prey model and puzzle box |

**Table S2:**

**Total monthly observation time for each type of observation and behavioral test.**

For each observation and behavioral test, the length of an individual observation bout, the number of observations conducted each month, the total observation length during each month, and how frequently the observation or behavioral test occurred. Quarterly indicates behavioral tests that occurred every three months.

| Observation/  Behavioral Test | Observation Bout length (min) | Bouts per month | Total time per month (min) | Frequency |
| --- | --- | --- | --- | --- |
| Focal observation (non-feeding) | 15 | 4 | 60 | Monthly |
| Focal observation (feeding w/o prey model) | 15 | 4 | 60 | Monthly |
| Focal observation (feeding w/ prey model) | 15 | 4 | 60 | Monthly |
| Novel object | 60 | 1 | 60 | Quarterly |
| Puzzle box | 120 | 10 | 1200 | Quarterly |
| Conspecific | 60 | 1 | 60 | Quarterly |

**Table S3:**

**Information on captive coyotes on the study, including behavioral tests, and how each performed.**

Details about the captive coyotes used as treatment and control animals on this study. Information on when each behavioral test was first introduced and how frequently the test was conducted throughout the study is provided. The first interaction column indicates when the individual coyote first interacted with the behavioral test. Interactions included any investigation, pursuit, or capture of the prey model, approaching within 1m of the novel object, investigation or physical contact with the puzzle box, and approaching within 5m of the conspecific dummy. The regularly interacted column indicates whether or not the coyote interacted with the behavioral test in multiple sequential trials. NA in the last two columns indicates coyotes that never interacted with the particular behavioral test.

| Coyote | Treatment | Mate | Behavioral Test | Month introduced | Frequency | First interaction | Regularly interacted |
| --- | --- | --- | --- | --- | --- | --- | --- |
| 1600 | Hunt | 1623 | Hunting prey model | January | Every week | September | No |
|  |  |  | Novel object (1m) (1m) | Pre-trial | Every 3 months | NA | NA |
|  |  |  | Puzzle box (int) | Pre-trial | Every 3 months | November | No |
|  |  |  | Conspecific | Pre-trial | Every 3 months | November | No |
|  |  |  |  |  |  |  |  |
| 1623 | Hunt | 1600 | Hunting prey model | January | Every week | NA | NA |
|  |  |  | Novel object (1m) | Pre-trial | Every 3 months | NA | NA |
|  |  |  | Puzzle box (int) | Pre-trial | Every 3 months | NA | NA |
|  |  |  | Conspecific | Pre-trial | Every 3 months | November | No |
|  |  |  |  |  |  |  |  |
| 1601 | Control | 1622 | Hunting prey model | January | Every week | July | Yes |
|  |  |  | Novel object (1m) | Pre-trial | Every 3 months | August | No |
|  |  |  | Puzzle box (int) | Pre-trial | Every 3 months | August | No |
|  |  |  | Conspecific | Pre-trial | Every 3 months | August | Yes |
|  |  |  |  |  |  |  |  |
| 1622 | Control | 1601 | Hunting prey model | January | Every week | NA | NA |
|  |  |  | Novel object (1m) | Pre-trial | Every 3 months | NA | NA |
|  |  |  | Puzzle box (int) | Pre-trial | Every 3 months | NA | NA |
|  |  |  | Conspecific | Pre-trial | Every 3 months | NA | NA |
|  |  |  |  |  |  |  |  |
| 1602 | Hunt | 1617 | Hunting prey model | January | Every week | April | No |
|  |  |  | Novel object (1m) | Pre-trial | Every 3 months | NA | NA |
|  |  |  | Puzzle box (int) | Pre-trial | Every 3 months | August | No |
|  |  |  | Conspecific | Pre-trial | Every 3 months | Pre-trial | No |
|  |  |  |  |  |  |  |  |
| 1617 | Hunt | 1602 | Hunting prey model | January | Every week | November | No |
|  |  |  | Novel object (1m) | Pre-trial | Every 3 months | NA | NA |
|  |  |  | Puzzle box (int) | Pre-trial | Every 3 months | NA | NA |
|  |  |  | Conspecific | Pre-trial | Every 3 months | August | Yes |
|  |  |  |  |  |  |  |  |
| 1610 | Control | 1703 | Hunting prey model | January | Every week | August | No |
|  |  |  | Novel object (1m) | Pre-trial | Every 3 months | NA | NA |
|  |  |  | Puzzle box (int) | Pre-trial | Every 3 months | NA | NA |
|  |  |  | Conspecific | Pre-trial | Every 3 months | August | No |
|  |  |  |  |  |  |  |  |
| 1703 | Control | 1610 | Hunting prey model | January | Every week | NA | NA |
|  |  |  | Novel object (1m) | Pre-trial | Every 3 months | NA | NA |
|  |  |  | Puzzle box (int) | Pre-trial | Every 3 months | NA | NA |
|  |  |  | Conspecific | Pre-trial | Every 3 months | August | No |
|  |  |  |  |  |  |  |  |
| 1802 | Hunt | 1853 | Hunting prey model | January | Every week | NA | NA |
|  |  |  | Novel object (1m) | Pre-trial | Every 3 months | NA | NA |
|  |  |  | Puzzle box (int) | Pre-trial | Every 3 months | November | No |
|  |  |  | Conspecific | Pre-trial | Every 3 months | Pretrial | No |
|  |  |  |  |  |  |  |  |
| 1853 | Hunt | 1802 | Hunting prey model | January | Every week | February | Yes |
|  |  |  | Novel object (1m) | Pre-trial | Every 3 months | NA | NA |
|  |  |  | Puzzle box (int) | Pre-trial | Every 3 months | NA | NA |
|  |  |  | Conspecific | Pre-trial | Every 3 months | Pre-trial | No |
|  |  |  |  |  |  |  |  |
| 1803 | Control | 1850 | Hunting prey model | January | Every week | NA | NA |
|  |  |  | Novel object (1m) | Pre-trial | Every 3 months | November | No |
|  |  |  | Puzzle box (int) | Pre-trial | Every 3 months | NA | NA |
|  |  |  | Conspecific | Pre-trial | Every 3 months | August | Yes |
|  |  |  |  |  |  |  |  |
| 1850 | Control | 1803 | Hunting prey model | January | Every week | May | Yes |
|  |  |  | Novel object (1m) | Pre-trial | Every 3 months | NA | NA |
|  |  |  | Puzzle box (int) | Pre-trial | Every 3 months | NA | NA |
|  |  |  | Conspecific | Pre-trial | Every 3 months | August | No |
|  |  |  |  |  |  |  |  |
| 1820 | Hunt | 1841 | Hunting prey model | January | Every week | April | Yes |
|  |  |  | Novel object (1m) | Pre-trial | Every 3 months | Pre-trial | Yes |
|  |  |  | Puzzle box (int) | Pre-trial | Every 3 months | Pre-trial | Yes |
|  |  |  | Conspecific | Pre-trial | Every 3 months | Pre-trial | Yes |
|  |  |  |  |  |  |  |  |
| 1841 | Hunt | 1820 | Hunting prey model | January | Every week | NA | NA |
|  |  |  | Novel object (1m) | Pre-trial | Every 3 months | NA | NA |
|  |  |  | Puzzle box (int) | Pre-trial | Every 3 months | Pre-trial | No |
|  |  |  | Conspecific | Pre-trial | Every 3 months | Pre-trial | No |
|  |  |  |  |  |  |  |  |
| 1842 | Control | 1851 | Hunting prey model | January | Every week | September | Yes |
|  |  |  | Novel object (1m) | Pre-trial | Every 3 months | May | No |
|  |  |  | Puzzle box (int) | Pre-trial | Every 3 months | NA | NA |
|  |  |  | Conspecific | Pre-trial | Every 3 months | NA | NA |
|  |  |  |  |  |  |  |  |
| 1851 | Control | 1842 | Hunting prey model | January | Every week | June | Yes |
|  |  |  | Novel object (1m) | Pre-trial | Every 3 months | NA | NA |
|  |  |  | Puzzle box (int) | Pre-trial | Every 3 months | Pre-trial | No |
|  |  |  | Conspecific | Pre-trial | Every 3 months | Pre-trial | Yes |

**Table S4:**

**Model output for non-feeding observations.**

Mixed-effects beta regression modeling results for the proportion of time spent resting, moving, feeding, and stereotyping during nonfeeding observations. Hunter indicates the main effect of the prey model treatment, month indicates the temporal trend, and hunter:month indicates the interaction effect.

| **Parameter** | **Est.** | **SE** | **z** | **p** |
| --- | --- | --- | --- | --- |
| *Resting* |  |  |  |  |
| Intercept | -0.060 | 0.18 | -0.33 | 0.743 |
| Hunter | -0.488 | 0.60 | -1.88 | 0.060 |
| Month | 0.047 | 0.02 | 2.28 | 0.022 |
| Hunter:month | 0.060 | 0.03 | 2.10 | 0.036 |
| *Moving* |  |  |  |  |
| Intercept | -1.354 | 0.16 | -8.59 | <0.001 |
| Hunter | 0.389 | 0.22 | 1.78 | 0.075 |
| Month | -0.059 | 0.02 | -3.46 | 0.001 |
| Hunter:month | -0.056 | 0.02 | -2.30 | 0.021 |
| *Feeding* |  |  |  |  |
| Intercept | -2.350 | 0.14 | -16.98 | <0.001 |
| Hunter | 0.072 | 0.18 | 0.41 | 0.685 |
| Month | -0.028 | 0.02 | -1.80 | 0.071 |
| Hunter:month | -0.017 | 0.02 | -0.78 | 0.440 |
| *Stereotyping* |  |  |  |  |
| Intercept | -3.982 | 0.14 | -27.86 | <0.001 |
| Hunter | 0.084 | 0.16 | 0.55 | 0.586 |
| Month | -0.002 | 0.02 | -0.15 | 0.885 |
| Hunter:month | -0.012 | 0.02 | -0.55 | 0.580 |

**Table S5:**

**Model output for feeding observations without the prey model present.**

Mixed-effects beta regression modeling results for the proportion of time spent feeding and moving during feeding observations. Hunter indicates the main effect of the prey model treatment, month indicates the temporal trend, and hunter:month indicates the interaction effect.

| **Parameter** | **Est.** | **SE** | **z** | **p** |
| --- | --- | --- | --- | --- |
| *Feeding* |  |  |  |  |
| Intercept | 0.248 | 0.16 | 1.59 | 0.112 |
| Hunter | -0.030 | 0.22 | -0.14 | 0.892 |
| Month | 0.041 | 0.01 | 3.03 | 0.002 |
| Hunter:month | 0.025 | 0.02 | 1.31 | 0.191 |
| *Moving* |  |  |  |  |
| Intercept | -1.054 | 0.14 | -7.54 | <0.001 |
| Hunter | -0.223 | 0.20 | -1.12 | 0.261 |
| Month | -0.101 | 0.01 | -8.61 | <0.001 |
| Hunter:month | -0.001 | 0.02 | -0.04 | 0.972 |

**Table S6:**

**Model output for feeding observations with the prey model present.**

Mixed-effects beta regression modeling results for the proportion of time spent feeding, moving, and interacting with the prey model during feeding observations with the prey model. Hunter indicates the main effect of the prey model treatment, month indicates the temporal trend, and hunter:month indicates the interaction effect.

| **Parameter** | **Est.** | **SE** | **z** | **p** |
| --- | --- | --- | --- | --- |
| *Feeding* |  |  |  |  |
| Intercept | -1.061 | 0.28 | -3.74 | <0.001 |
| Hunter | 0.419 | 0.40 | 1.04 | 0.299 |
| Month | 0.014 | 0.02 | 0.81 | 0.417 |
| Hunter:month | -0.100 | 0.03 | -3.95 | <0.001 |
| *Moving* |  |  |  |  |
| Intercept | -0.170 | 0.11 | -1.50 | 0.134 |
| Hunter | -0.527 | 0.16 | -3.28 | 0.001 |
| Month | -0.111 | 0.01 | -7.76 | <0.001 |
| Hunter:month | 0.082 | 0.02 | 4.11 | <0.001 |
| *Interacting with prey model* |  |  |  |  |
| Intercept | -4.635 | 0.25 | -18.45 | <0.001 |
| Hunter | -0.104 | 0.33 | -0.32 | 0.753 |
| Month | 0.040 | 0.02 | 2.39 | 0.017 |
| Hunter:month | 0.059 | 0.02 | 2.52 | 0.012 |

**Table S7:**

**Model output for latency to approach the novel object.**

Mixed effects Cox proportional hazards modeling results for the latency to approach within 5m and 1m of the novel object. Hunter indicates the main effect of the prey model treatment, month indicates the temporal trend, and hunter:month indicates the interaction effect.

| **Parameter** | **Est.** | **SE** | **z** | **p** |
| --- | --- | --- | --- | --- |
| *5m* |  |  |  |  |
| Hunter | 0.213 | 1.06 | 0.20 | 0.840 |
| Month | 0.316 | 0.19 | 1.69 | 0.090 |
| Hunter:month | -0.050 | 0.26 | -0.18 | 0.860 |
| *1m* |  |  |  |  |
| Hunter | 1.246 | 2.70 | 0.46 | 0.650 |
| Month | 0.524 | 0.47 | 1.12 | 0.260 |
| Hunter:month | -0.401 | 0.57 | -0.70 | 0.490 |

**Table S8:**

**Model output for proportion of time spent near and interacting with the puzzle box.**

Mixed-effects beta regression modeling results for the proportion of time spent within 5m, 1m, and interacting with the puzzle box. Hunter indicates the main effect of the prey model treatment, month indicates the temporal trend, and hunter:month indicates the interaction effect.

| **Parameter** | **Est.** | **SE** | **z** | **p** |
| --- | --- | --- | --- | --- |
| *Proportion 5m* |  |  |  |  |
| Intercept | -3.747 | 0.26 | -14.39 | <0.001 |
| Hunter | -0.302 | 0.37 | -0.83 | 0.409 |
| Month | 0.105 | 0.04 | 3.03 | 0.002 |
| Hunter:month | 0.241 | 0.05 | 4.79 | <0.001 |
| *Proportion 1m* |  |  |  |  |
| Intercept | -4.718 | 0.26 | -18.27 | <0.001 |
| Hunter | -0.337 | 0.36 | -0.95 | 0.342 |
| Month | 0.084 | 0.04 | 2.29 | 0.022 |
| Hunter:month | 0.232 | 0.05 | 4.27 | <0.001 |
| *Proportion interacting* |  |  |  |  |
| Intercept | -6.372 | 0.17 | -36.83 | <0.001 |
| Hunter | -0.060 | 0.18 | -0.33 | 0.741 |
| Month | 0.002 | 0.04 | 0.06 | 0.953 |
| Hunter:month | 0.043 | 0.05 | 0.82 | 0.415 |

**Table S9:**

**Model output for latency to approach the puzzle box.**

Mixed effects Cox proportional hazards modeling results for the latency to approach within 5m, 1m, and interact with the puzzle box. Hunter indicates the main effect of the prey model treatment, month indicates the temporal trend, and hunter:month indicates the interaction effect.

| **Parameter** | **Est.** | **SE** | **z** | **p** |
| --- | --- | --- | --- | --- |
| *Latency 5m* |  |  |  |  |
| Hunter | 0.063 | 0.48 | 0.13 | 0.900 |
| Month | -0.066 | 0.06 | -1.09 | 0.280 |
| Hunter:month | 0.233 | 0.08 | 2.82 | 0.005 |
| *Latency 1m* |  |  |  |  |
| Hunter | 0.539 | 0.90 | 0.60 | 0.550 |
| Month | 0.205 | 0.07 | 2.78 | 0.006 |
| Hunter:month | 0.187 | 0.10 | 1.88 | 0.060 |
| *Latency to interact* |  |  |  |  |
| Hunter | -2.284 | 2.04 | -1.12 | 0.260 |
| Month | 0.176 | 0.41 | 0.43 | 0.670 |
| Hunter:month | 1.240 | 0.48 | 2.59 | 0.010 |

**Table S10:**

**Model results for the latency to approach the conspecific decoy.**

Mixed effects Cox proportional hazards modeling results for the latency to approach within 5m and 1m of the conspecific decoy.. Hunter indicates the main effect of the prey model treatment, month indicates the temporal trend, and hunter:month indicates the interaction effect.

| **Parameter** | **Est.** | **SE** | **z** | **p** |
| --- | --- | --- | --- | --- |
| *Latency 5m* |  |  |  |  |
| Hunter | 1.489 | 1.13 | 1.31 | 0.190 |
| Month | 0.429 | 0.21 | 2.00 | 0.045 |
| Hunter:month | -0.330 | 0.29 | -1.14 | 0.260 |
| *Latency 1m* |  |  |  |  |
| Hunter | 0.991 | 2.01 | 0.49 | 0.62 |
| Month | -0.455 | 0.38 | -1.19 | 0.23 |
| Hunter:month | 0.120 | 0.504 | 0.24 | 0.81 |

**Table S11:**

**PERMANOVA model results for each month testing for multivariate effects of hunting treatment on coyote behavior.**

| **Parameter** | **DF** | **Sums of Squares** | **R2** | **F** | **p** |
| --- | --- | --- | --- | --- | --- |
| *Pretrial* |  |  |  |  |  |
| Hunter | 1 | 7.855 | 0.091 | 1.407 | 0.242 |
| Residual | 14 | 78.139 |  |  |  |
| Total | 15 | 85.993 |  |  |  |
| *February* |  |  |  |  |  |
| Hunter | 1 | 3.801 | 0.082 | 1.253 | 0.298 |
| Residual | 14 | 42.48 |  |  |  |
| Total | 15 | 46.282 |  |  |  |
| *May* |  |  |  |  |  |
| Hunter | 1 | 5.625 | 0.064 | 0.825 | 0.605 |
| Residual | 12 | 81.836 |  |  |  |
| Total | 13 | 87.462 |  |  |  |
| *August* |  |  |  |  |  |
| Hunter | 1 | 17.772 | 0.085 | 1.297 | 0.237 |
| Residual | 14 | 191.869 |  |  |  |
| Total | 15 | 209.641 |  |  |  |
| *November* |  |  |  |  |  |
| Hunter | 1 | 22.001 | 0.223 | 4.024 | 0.018 |
| Residual | 14 | 76.537 |  |  |  |
| Total | 15 | 98.537 |  |  |  |
